# From ambition to substance use tendency: the mediating role of significance quest

**DOI:** 10.3389/fpsyg.2025.1496907

**Published:** 2025-03-07

**Authors:** Ekrem Sedat Şahin

**Affiliations:** Department of Educational Sciences, Faculty of Education, Aksaray University, Aksaray, Türkiye

**Keywords:** ambition, quest for sigficance, substance use tendency, emerging adults, structural equation modeling

## Abstract

In this study, the aim was to determine the mediating effect of the quest for significance on the relationship between ambition and substance use tendency in emerging adults. Data were collected from emerging adults studying at various universities in Turkey through both face-to-face and online processes. A total of 506 emerging adults ( $\bar{X}$ _age_ = 21.62) participated in the study. The Ambition Scale, Significance Quest Scale, Substance Initiation Tendency Scale, and a demographic form were used to gather data. Structural equation modeling was employed to analyze the data. The results revealed a positive and significant relationship between ambition and the quest for significance, as well as between the quest for significance and substance use tendency in emerging adults. Furthermore, the quest for significance was found to mediate the relationship between ambition and substance use tendency in this population. Suggestions were provided based on the findings of the study.

## Highlights

- Ambition may play a triggering role in the quest for significance among emerging adults.
- The quest for significance has a positive effect on the impulsive dimension of emerging adults’ tendency toward substance use.
- There is a positive effect of the quest for significance on the attitudinal dimension of emerging adults’ tendency toward substance use.
- Substance use and addiction can potentially be prevented by helping emerging adults feel a sense of importance.

## Introduction

Substance addiction has existed throughout human history as a challenging public health problem that poses risks to individuals at different stages of life, from adolescents to the elderly (Gray and Squeglia, 2018; Lo et al., 2020; Yarnell et al., 2020). Emerging adulthood is a particularly vulnerable period during which substance abuse and mental health problems are likely to escalate, posing substantial risks for the development of chronic conditions, such as substance addiction, that may affect young people later in life (Kirst et al., 2014). According to Arnett (2000, 2005), emerging adulthood, defined as the age range between 18 and 25, is marked by an intensified search for identity, career, and romantic relationships, as well as experimentation and decision-making about one’s role in the world. Epidemiological studies indicate that the prevalence of substance use increases during adolescence and peaks in emerging adulthood (Palmer et al., 2009). According to Turkish Statistical Institute (TURKSTAT) data for 2023, the emerging adult population in Turkey constitutes 12.2% of the total population (TURKSTAT, 2025). It can be said that research and prevention studies on substance use tendencies of emerging adults in Turkey are important for both individual and public health. While many emerging adults tend to “mature out” of substance use after successfully navigating key developmental transitions related to work and family, a considerable number of individuals fail to do so, leading to long-term physical and mental health issues (Jackson et al., 2008). Preventing the onset and progression of substance abuse in emerging adults is crucial for their current and future well-being. In this regard, identifying the factors that predispose emerging adults to substance use is of paramount importance.

### Substance use tendency

Substance use adversely affects not only the individual but also their family, friends, neighbors, and broader environment (Entezari and Saroogh Farahan, 2013). It is one of the most significant issues confronting emerging adults in the United States (Mochrie et al., 2020; Walters et al., 2018). According to a study conducted by the SAMHSA (2021), the most commonly used substances among Americans are alcohol, tobacco, and cannabis. SAMHSA (2021) data reveal that alcohol use has risen to 67.99%, tobacco and tobacco products to 27.26%, and drug use to 25.77% among American youth aged 18–25. In contrast, the Turkish Health Survey conducted by the (TURKSTAT) found that tobacco and tobacco product use among individuals aged 15 and over was 28.30%, while alcohol use in the same age group was 12.10% (TURKSTAT, 2022). A survey by the Security General Directorate of the Republic of Turkey in 2018, involving 1,338 participants, found that 3.10% reported having used drugs at least once in their lives, with 35.40% of these individuals aged 15–24 (Security General Directorate, 2018). According to the NCDAS (2020) data, alcohol is the most frequently used substance among young people aged 18–25 in the United States, with 11.72 million American emerging adults consuming alcohol. Conversely, 4,777 individuals in this demographic died from drug overdoses (NCDAS, 2020).

Substance abuse among emerging adults is not a new problem in Turkey as it is in the world. The concepts of individual and social welfare imposed by industrialization, modernization and urbanization have not always produced the expected results. Even though it has produced the desired results, it has also caused a process that isolates the individual and increases the number of problems he/she has to cope with. This process may cause some emerging adults to turn to substance use and addiction (Duyan and Gövebakan, 2021). Substance use in emerging adults can result in physical or psychological health problems, self-harm, suicide and even death. For emerging adults who are university students, substance use can exacerbate these issues and additionally lead to poor academic performance, including lower class attendance, missed classes, reduced study time, poor grades, failure to graduate, and unemployment after graduation (Buckner et al., 2010; Patterson et al., 2021; Skidmore et al., 2016; Welsh et al., 2019; Zhang et al., 2018). Furthermore, substance use among emerging adults can contribute to socialization problems, difficulties in interpersonal relationships, low energy, decreased motivation, memory issues, procrastination, and reduced productivity (Buckner et al., 2010; Mochrie et al., 2020; Walters et al., 2018). Scientific interest in understanding the risk factors underlying substance use and related disorders is growing rapidly. Many researchers have focused on identifying common risk factors across different substances due to the high comorbidity rates between nicotine, alcohol, and drugs (Palmer et al., 2009). Emerging adults encounter unique stressors that can impact their mental health and lead to problematic substance use (Metzger et al., 2017). A study on emerging adults who are university students in Turkey found that individuals in this age group experience economic difficulties, academic problems, and difficulties in personal adjustment and social relationships (Donat et al., 2019). It is common for individuals in this age group to attempt to cope with elevated stress levels through self-medication using unhealthy methods such as smoking, alcohol, or drug use (Patterson et al., 2021). Peer influence, the perception that a substance is harmless or has minimal harm, poor academic performance, and club membership among university students are notable risk factors for substance use (Welsh et al., 2019). Interpersonal relationships are also effective in substance use and addiction. Individuals who experience social exclusion in their relationships feel insignificant and this perception of insignificance may increase the risk of substance abuse (de Espíndola et al., 2020; Flett et al., 2023). Elliott (2009) noted that substance use can serve as an escape for those who feel insignificant. The need to feel significant is a fundamental human need, similar to other basic needs such as biological ones. This need can be situationally activated and can influence behavior. When an individual feels insignificant, the need for significance may become more pronounced, potentially leading to a quest for significance (Kruglanski et al., 2022). In other words, the quest for significance can also drive individuals toward substance use (Flett et al., 2023).

### Quest for significance

Mattering, considered one of the fundamental human needs, is conceptualized as a personal resource. Individuals who feel that they matter experience a sense of security through meaningful connections and close social relationships. Conversely, those who perceive themselves as insignificant may experience unhappiness, vulnerability, and anger (Flett, 2018; Flett et al., 2021; Flett et al., 2022; Taylor and Turner, 2001). According to Rosenberg and McCullough (1981), our perception of being important is reflected in our belief that others are connected to us, are interested in our future, or see us as an extension of their own ego. This perception is thought to stem from four sources: attention, importance, dependence, and ego-extension.

- Attention refers to our awareness that our actions are noticed by others.
- Importance denotes the perception that our actions are significant to others.
- Dependence arises from the social connections and obligations between individuals.
- Ego-extension involves the perception that others have an emotional investment in us, will miss us if we leave, or that our successes or failures will elicit joy or disappointment from those around us (Rosenberg and McCullough, 1981; Taylor and Turner, 2001).

Perceptions of self-importance in the eyes of others arise from social interactions that foster a sense of belonging, identity, and commitment (Taylor and Turner, 2001). Individuals who desire to be viewed as ‘positive’ and ‘valuable’ by those with whom they interact are driven by fundamental needs to be valued, admired, and considered important (Kruglanski et al., 2022). The concept of a quest for significance refers to the individual’s need to be important and respected both in the eyes of others and in their own self-perception (Kruglanski et al., 2009; Kruglanski and Bertelsen, 2020). This concept is explained by Significance Quest Theory (SQT), which posits that the need to feel cared for is a basic and universal human requirement. It encompasses the desire to feel valuable and important and to perceive oneself as significant within one’s social context (Contu et al., 2023; Kruglanski et al., 2022). SQT suggests that people are motivated to seek this sense of significance when they feel deprived of it, and they plan and manifest their actions to achieve a sense of importance. The theory identifies two types of motivations for seeking significance: collective quest for significance, which arises from perceptions of one’s group being humiliated or disrespected, and individual quest for significance, triggered by personal experiences (Schumpe et al., 2020; Jasko et al., 2020).

The experience of losing significance is often linked to one’s social identity being disrespected by others (Kruglanski et al., 2022). When a group is humiliated or its sacred values are violated, all members of the group can experience a profound sense of loss of significance (Kruglanski et al., 2014). On the other hand, the quest for individual significance is triggered by personal experiences of humiliation unrelated to the group or society in which the person lives. Failures at school or work, rejection by a desirable individual, stigmatization (such as infertility, divorce, or extramarital affairs), economic loss, or victimization due to war or natural disasters can all lead to a loss of individual significance. These experiences typically make a person feel powerless and unimportant (Kruglanski and Bertelsen, 2020; Kruglanski et al., 2014). Experiences such as exclusion, discrimination, disenfranchisement, insults, failure, and defeat can lead to feelings of humiliation, indignity, dishonor, and shame. These negative experiences contribute to the perception of a loss of significance in the individual (Kruglanski et al., 2023).

When the quest for significance is prioritized over other current anxieties, it drives action toward achieving significance (Kruglanski et al., 2022). However, individuals do not constantly pursue significance. The quest for significance can vary both within and between individuals. In other words, it is not always at the forefront of one’s mind (Contu et al., 2023). Even the most passionate idealists do not continuously seek significance; they also engage in self-protective behaviors, addressing needs such as physiological requirements, safety, and comfort (Kruglanski et al., 2013). According to the Significance Quest Theory (SQT), the quest for significance occurs under three conditions: an individual’s loss of significance, the perceived threat of losing current significance, and the opportunity to gain significance (Kruglanski et al., 2014; Kruglanski et al., 2022; Kruglanski and Bertelsen, 2020). SQT argues that when people experience a loss of significance due to humiliation, disrespect, rejection, and failure, or when they perceive a threat to the loss of significance, they tend to find the necessary means to regain significance (Jasko et al., 2020; Kruglanski et al., 2013; Kruglanski et al., 2014; Webber et al., 2017). The loss or risk of losing significance disrupts the individual’s motivational balance, prompting them to search for significance to restore equilibrium. This quest for significance, in this context, is viewed as a preventive measure by Kruglanski et al. (2022). On the other hand, the quest for significance can also be triggered by various opportunities to gain significance, referred to as ‘appetizers’ (Kruglanski et al., 2023). Such opportunities can arise in the context of a loss of significance (Kruglanski et al., 2015) or independently, without any prior loss.

The literature on the quest for significance indicates that even brief experiences of humiliation or failure can drive individuals to take extreme actions to regain their lost significance (Kruglanski et al., 2022). These actions may include substance abuse. When an individual’s sense of significance is threatened, especially for those with high ambition, they may be more willing to make significant sacrifices for a worthy goal to restore their prestige and reputation. Ambitious individuals are characterized by a strong motivation toward achieving substantial and meaningful goals, including gaining respect and appreciation from others. They are more sensitive to threats against their perception of significance (Resta et al., 2023a). Those who achieve social significance often possess various skills, abilities, and characteristics, but a common trait among them is ambition. Ambitious individuals are more likely to achieve the sense of significance they seek by being ‘the best’ in their respective fields (Resta et al., 2022).

### Ambition

Ambition is a frequently discussed concept in social science research but remains insufficiently understood (Judge and Kammeyer-Mueller, 2012). It is a construct with neurobiological origins, linked to factors such as testosterone levels and dopamine. The expression of ambition is also influenced by culture, social class, and gender (Yager and Kay, 2023). Ambition represents the desire to achieve goals. Sociologists view ambition as a product of parental behavior and socio-economic conditions, while psychologists consider it a personality trait (Mahmood and Alwan, 2022). Ambition is defined as an intermediate personality trait and an aspect of extraversion. It can also be summarized as the goal of gaining respect and recognition from others (Judge and Kammeyer-Mueller, 2012; Resta et al., 2023b). As a personality construct, ambition has significant implications for individual differences in educational and career success, as well as status attainment. Ambitious individuals are characterized by their energy, status-seeking behavior, determination to achieve their goals, unwavering focus on their mission, and minimal tendency toward procrastination (Jones et al., 2017). Ambitious individuals quest for the highest level of significance and are therefore likely to place less value on the rewards provided by their immediate networks. Instead, they focus on surpassing their local network and maximizing their overall significance (Ellenberg and Kruglanski, 2024). Ambition is marked by a strong commitment to achieving worthwhile and impressive goals. Consequently, ambitious people are not merely satisfied with attaining status; they actively select and employ specific strategies to stand out and gain recognition. Compared to less ambitious individuals, those with high ambition are more inclined to engage in self-enhancing behaviors (Resta et al., 2023b).

Ambition is viewed as a complex characteristic of human personality that encompasses both ‘having’ and ‘realizing’ dimensions (Judge and Kammeyer-Mueller, 2012). It explains how individuals relate to their environment in order to change and develop, enabling them to be prepared for goals, engage in struggles, take responsibility, and devise and implement strategies (Mahmood and Alwan, 2022). In this sense, ambition is an active trait that shapes both present and future behaviors. It enhances individuals’ action processes, thinking, skills, and practices in a way that fosters confidence (Alshebami and Alamri, 2020). Ambition, characterized by its generality and continuity, is not solely assessed by success in one area and does not diminish once a particular goal is achieved. Instead, ambition is more about the pursuit of success than merely achieving it (Judge and Kammeyer-Mueller, 2012). Ambition is the driving force that enables a person to advance, be promoted, and ascend the social ladder. It plays a crucial role in self-actualization both professionally and in life more broadly. An ambitious person actively applies their potential in socially significant areas (Barsukova et al., 2015). As a motivational trait, ambition represents the individual’s desire to achieve meaningful accomplishments that matter both to themselves and to others, and to be recognized and valued by others (Barsukova, 2014). There is a positive and significant relationship between ambition and taking responsibility (El Baroudi et al., 2017). Ambitious individuals strive for success, seek popularity, fame, and power, and aim to enhance their status and position in the world (Barsukova et al., 2015). At the core of ambition lies the need for self-affirmation and recognition, alongside greed, which drives all desire. These motives lead individuals to seek admiration and attention, thereby feeding narcissistic tendencies. Ambition is viewed as both a “good” and a “bad” trait (Barsukova, 2015). On one hand, good ambition provides meaning and direction to an individual’s life, contributing significantly through achievements and personal fulfillment. It can enhance moral development by fostering creativity, productivity, discipline, and perseverance (Pettigrove, 2007). On the other hand, bad ambition can distort personality development and interpersonal relationships. It may lead to disappointments, negative emotions, and the development of aggressive or dependent behaviors (Barsukova, 2015). When evaluating ambition as “good” or “bad,” it is important to consider both the value of the object of ambition and the intensity of the emotions it generates. Most objects of ambition involve competition and are unlikely to be achieved quickly, regardless of how strongly they are desired. Success in achieving ambitious goals typically requires self-discipline, commitment, and determination (Pettigrove, 2007).

Ambition drives individuals to advance their careers and grow professionally. In this regard, ambition steers behavior toward socially accepted and significant achievements (Barsukova, 2014). Ambitious individuals exhibit high achievement motivation. Their basic self-regulation and cognitive characteristics support goal attainment. While generally maintaining a positive self-attitude, ambitious individuals may occasionally experience dissatisfaction with their achievements. Their competitive nature and tendency to compare themselves with others can impact their social relationships. Additionally, ambitious individuals are focused on intense work and minimizing idle time. They may be willing to change jobs to build a career and reach the professional “top.” Those who seek recognition from others pursue their goals actively and consistently (Barsukova, 2016; Barsukova et al., 2015). Ambition, as a personality trait, is related to the quest for significance, a universal human need, and is also thought to be connected to a tendency toward substance abuse.

### Current research

Ambition is related to constructs such as achievement, desire for power, conscience, self-esteem, perception of relative deprivation, and the quest for significance (Resta et al., 2023b). Thus, ambition can be conceptualized as a heightened sensitivity to opportunities for gaining significance or a desire to maximize one’s significance, rather than settling for a ‘good enough’ amount of it (Ellenberg and Kruglanski, 2024; Kruglanski et al., 2013; Resta et al., 2022). Greenberg et al. (2004) found that ambitious employees are more likely to take on responsibilities to achieve a greater sense of job significance. Similarly, Barrick et al. (2013) noted that extroverted and ambitious employees often experience a strong sense of significance in their work. Resta et al. (2023b) discovered that ambitious individuals are particularly sensitive to disappointments that threaten their sense of significance. Consequently, the decision was made to examine the relationship between ambition and the quest for significance within a sample of emerging adults.

There is substantial evidence linking feelings of insignificance, which underlie the quest for significance, to substance abuse and addictive tendencies (Flett et al., 2023). Fernandez et al. (2009) found that drug use is more prevalent among individuals experiencing problems with social acceptance. Edwards and Neal (2017) found a statistically significant negative relationship between the feeling of mattering and excessive alcohol consumption. Similarly, Schmidt (2018) found a significant negative relationship between the feeling of significance and substance abuse. Edwards et al. (2021) found that students’ perception of insignificance at school is related to alcohol use. Additionally, Bahl et al. (2023) found a negative relationship between mattering and substance use in a sample of older adults. In this context, individuals who feel insignificant and subsequently quest for significance may also be more prone to substance use. Trucco (2020) suggests that the choice of friends and socialization play a significant role in substance use. Especially during adolescence and emerging adulthood, substance use is influenced by both peer selection and socialization processes with peers. One factor that can trigger the quest for significance in individuals is the opportunity to gain importance (Kruglanski et al., 2023). In this context, emerging adults’ substance use may be an attempt to join a group, gain acceptance, or emulate group members. In other words, substance use might serve as a means to achieve some form of significance. As previously mentioned, emerging adulthood is a particularly risky period for substance use (Arnett, 2005; Kirst et al., 2014). Therefore, examining the relationship between the quest for significance and substance abuse tendencies in emerging adults is expected to provide valuable insights for prevention strategies related to substance use and addiction. The following hypotheses were tested in the study.

*H1:* There is a positive and significant relationship between ambition and the quest for significance in emerging adults.

*H2:* There is a positive and significant relationship between the quest for significance and the impulsivity dimension of substance abuse tendencies in emerging adults.

*H3:* There is a positive and significant relationship between the quest for significance and the attitude dimension of substance abuse tendencies in emerging adults.

*H4:* The quest for significance mediates the relationship between ambition and the drive dimension of substance abuse tendencies in emerging adults.

*H5:* The quest for significance mediates the relationship between ambition and the attitude dimension of substance abuse tendencies in emerging adults.

## Method

### Research desing

In this study, the correlational survey model was employed to assess the mediating role of the quest for significance in the relationship between ambition and substance use tendencies in emerging adults. The correlational survey model is used to determine the presence and/or degree of covariance between two or more variables (Creswell, 2014).

### Participants

In this study, data were collected using convenience sampling. Convenience sampling is a non-probability sampling method that involves collecting data from participants who are most accessible to the researcher, willing to participate, or otherwise easily reachable (Scholtz, 2021). In this study, 506 emerging adults ( $\bar{X}$ _age_ = 21.62) studying at a university in Turkey participated. Of the participants, 369 (72.9%) identified as female, 134 (26.5%) identified as male, and 3 (0.6%) chose not to specify their gender. In the study, information on the age of the participants was collected; information on their grade level was not collected. In terms of age, 66 (13%) were 19 years old or younger, 200 (39.5%) were between 20 and 21 years old, 152 (30.0%) were between 22 and 23 years old, and 88 (17.4%) were 24 years old or older.

### Data collection and ethical considerations

The research was conducted in accordance with the decision numbered 2024/02–161 from the Human Research Ethics Committee of a state university in Turkey. Data for the study were collected through both face-to-face and online processes. In both processes, participants were presented with an Informed Consent Form and were informed about the confidentiality of the data, as well as the purpose and significance of the research. This information aimed to encourage emerging adults to provide sincere and honest responses to the measurement tools. Additionally, in the face-to-face process, individuals who were hesitant about participating were not pressured, and only those who voluntarily chose to participate were included. In the online process, participants who indicated their voluntary participation by marking ‘Yes’ were included in the study.

### Data collection tools

#### Ambition scale (AS)

This scale was developed by Hirschi and Spurk (2021) and adapted to Turkish culture by Şahin and Ayvaz (2024). The one-dimensional, five-item AS includes items such as “*I’m ambitious.”* and “*It is very important for me to achieve extraordinary results in my life*.” The Cronbach’s alpha value reported in the adaptation study was 0.78. The correlation coefficient from the test–retest application was 0.72. The scale demonstrated a good fit to the data based on the goodness of fit values calculated in the Confirmatory Factor Analysis (CFA) [χ^2^(4) = 12.69, *p* = 0.013, CFI = 0.98, TLI = 0.96, SRMR = 0.03, RMSEA = 0.08, 90% CI (0.03, 0.13)] (Şahin and Ayvaz, 2024). In this study, the Cronbach’s alpha value of the scale was recalculated and found to be 0.82.

#### Significance quest scale (SQS)

This scale was developed by Şahin and Derin (2023) and consists of four sub-dimensions and 26 items. The scale includes items such as “*I try to show my difference somehow.*” and “*I do my best to impress everyone around me.*” Exploratory Factor Analysis (EFA) and Confirmatory Factor Analysis (CFA) were conducted during the development process of the scale. The goodness of fit values obtained from the CFA were χ^2^/df = 1.89, RMSEA = 0.065, GFI = 0.86, IFI = 0.91, TLI = 0.92, and CFI = 0.92. The Cronbach’s alpha coefficient, which measures the internal consistency of the scale, was 0.95, and the test–retest correlation coefficient was 0.84 (Şahin and Derin, 2023). In this study, the Cronbach’s alpha value of the scale was recalculated and found to be 0.94.

#### Substance initiation tendency scale (SITS)

This scale, developed by Önder (2023), consists of two sub-dimensions and eight items. The scale includes items such as “*I wonder what the substance is like.*” and “*Sometimes something inside me pushes me to use the substance.”* Both Exploratory Factor Analysis (EFA) and Confirmatory Factor Analysis (CFA) were conducted during the development process of the SITS. The CFA results indicated a good fit to the data, with the following values: *χ*^2^/df = 2.99, RMSEA = 0.06, GFI = 0.97, AGFI = 0.94, IFI = 0.96, NFI = 0.94, TLI = 0.94, and CFI = 0.96. The Cronbach’s alpha coefficient, which measures the internal consistency of the scale, was 0.80 (Önder, 2023). In this study, the Cronbach’s alpha value of the SITS was recalculated and found to be 0.80.

#### Personal information form (PIF)

The PIF was developed by the author. The form includes questions to determine the gender and age of the participants.

### Data analysis

SPSS 27.0 was used to test the assumptions in the data set and to conduct preliminary analyses. Structural equation modeling was performed using AMOS 24.0 with the maximum likelihood estimation method to test the research hypotheses. Model goodness-of-fit evaluations were based on the *χ*^2^/df, CFI, RMSEA, and SRMR indices. Acceptable goodness-of-fit values were determined as *χ*^2^/df < 5, CFI > 0.90, RMSEA <0.08, and SRMR <0.08, as widely referenced (Anderson and Gerbing, 1988; Hu and Bentler, 1999; Keith, 2019). In constructing the latent variables in the model, scale items for the ambition scale and substance abuse tendency scales were treated as observed variables. Since the significance quest variable is a multi-item and multidimensional construct, the total scores of the sub-dimensions of the Significance Quest Scale were used as observed variables when constructing the latent variable. Finally, hypotheses regarding indirect relationships in the model were tested using bootstrap percentile confidence intervals (Tibbe and Montoya, 2022).

### Results

**Figure 1:**
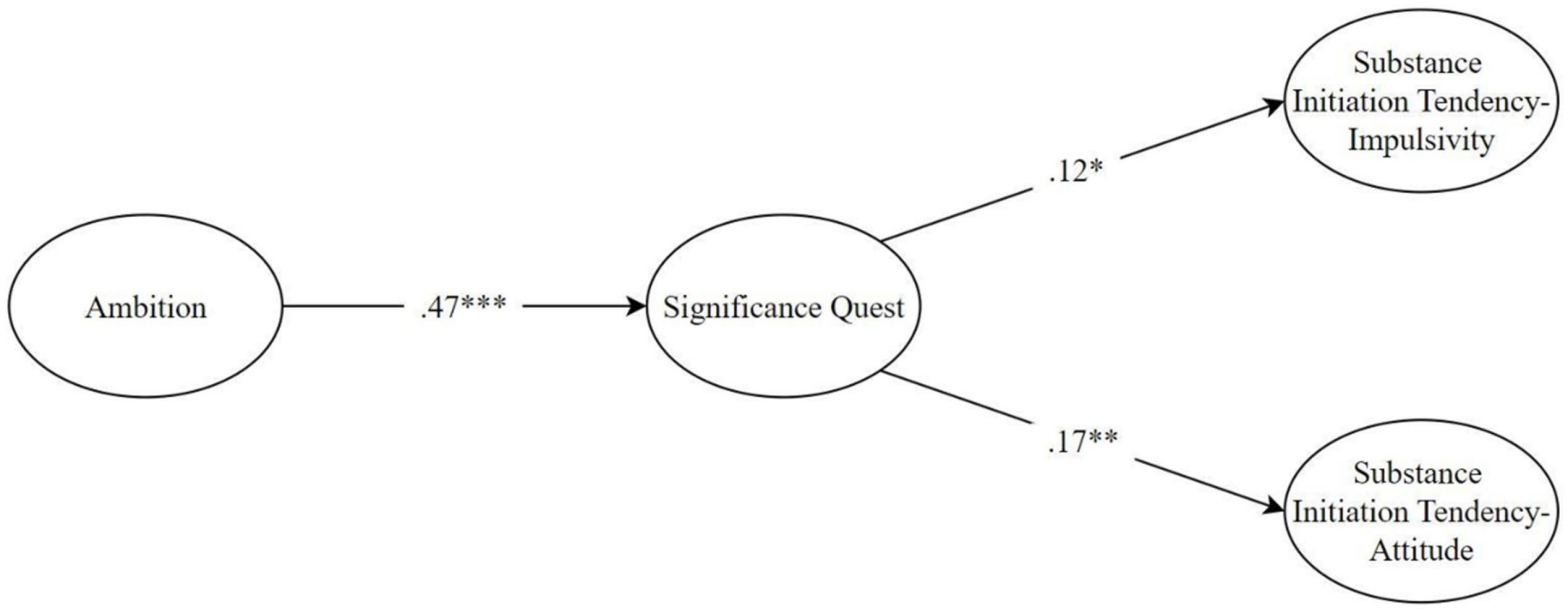
Structural model with standardized path estimates.

Firstly, the goodness of fit of the measurement model was tested. The goodness-of-fit values for the measurement model were found to be acceptable [*χ*^2^(113) = 330.27, *p* < 0.001, *χ*^2^/df = 2.92, CFI = 0.93, RMSEA = 0.062, 90% CI (0.054, 0.070), SRMR = 0.048]. Item factor loadings ranged from 0.18 to 0.86. One of the items belonging to the attitude dimension of the Substance Initiation Tendency Scale has a factor loading of 0.18 and weakly represents the relevant factor. However, when the content of the item is analyzed, it is thought that it is theoretically important and strongly represents the construct in terms of content. As Hair et al. (2019) stated, in the case of items that are considered to represent the construct well from a theoretical perspective, the relevant item can be retained in the measurement model even if there is no strong evidence of construct validity from a statistical perspective. For this reason, the item was kept in the measurement model considering both the theoretically good representation of the item in the attitude dimension of the tendency to use substances and the good values in the model fit indices. After validating the measurement model, the goodness of fit of the structural model, created in line with the research hypotheses, was examined as shown in Figure 1. The goodness-of-fit values for the structural model were also found to be acceptable [*χ*^2^(115) = 309.10, *p* < 0.001, *χ*^2^/df = 2.95, CFI = 0.93, RMSEA = 0.062, 90% CI (0.055, 0.070), SRMR = 0.056].

After validating the structural model, we analyzed the results regarding the direct relationships. Ambition significantly predicted the quest for significance positively (*β* = 0.47, B = 2.13, SE = 0.27, *p* < 0.001). The quest for significance significantly predicted the impulse dimension of substance use tendency positively (*β* = 0.12, B = 0.01, SE = 0.01, *p* = 0.020). Similarly, the quest for significance significantly predicted the attitude dimension of substance use tendency positively (*β* = 0.17, *B* = 0.02, SE = 0.01, *p* = 0.002).

After analyzing the direct relationships, we tested indirect correlations, as detailed in Table 1. These indirect correlations were assessed using bootstrap percentile confidence intervals based on 10,000 samples. The results indicated that the quest for significance significantly mediated the relationship between ambition and the drive dimension of substance use tendency [*β* = 0.08, SE = 0.03, 95% CI (0.03, 0.13)]. Similarly, the quest for significance significantly mediated the relationship between ambition and the attitude dimension of substance use tendency [*β* = 0.06, SE = 0.03, 95% CI (0.01, 0.10)].

**Table 1:** Tests of indirect effects.

Predictor	Mediator	Outcome	Standardized indirect effect *β*	Standard errors	Percentile confidence intervals [95%]
Ambition	Significance quest	Substance initiation tendency-impulsivity	0.06	0.03	[0.01, 0.10]
Ambition	Significance quest	Substance initiation tendency-attitude	0.08	0.03	[0.03, 0.13]

## Discussion and interpretations

In the study, it can be said that there is a moderate, positive and significant relationship (*β* = 0.47) between ambition and quest for significance in emerging adults and accordingly, hypothesis H_1_ is supported. This finding is consistent with results reported in previous research by Greenberg et al. (2004), Barrick et al. (2013), and Resta et al. (2023b). Ambition, defined as the drive to achieve goals and gain respect and recognition from others, reflects an individual’s pursuit of status and career advancement. Highly ambitious individuals are motivated to reach their objectives and attain prominence within their professional fields. They actively and persistently seek recognition and may feel dissatisfied with the opportunities for significance available within their immediate social networks. Such individuals are often highly sensitive to incentives related to gaining significance and strive to enhance their sense of importance beyond what is merely “good enough,” aiming instead for “much better.” On the other hand, ambitious individuals are also more sensitive to the potential loss of significance (Barsukova, 2016; Bertelsen and Kruglanski, 2024; Jones et al., 2017; Judge and Kammeyer-Mueller, 2012; Resta et al., 2023b). According to Resta et al. (2022), ambition, defined as the desire for recognition and respect, can be conceptualized as a form of the quest for significance. Ambitious individuals who experience a decrease in significance or perceive a threat to their current level of significance are likely to engage in this quest more readily than their less ambitious counterparts. Those striving for better conditions and higher status are more adept at identifying and capitalizing on opportunities to gain significance, thus leveraging these opportunities to enhance their importance in both their own eyes and those of others. In summary, ambition, as an aspect of extraversion and an intermediate personality trait, may positively catalyze the quest for significance, which in turn can facilitate the attainment of goals for ambitious individuals.

Ambition, considered a neurobiologically based personality trait (Yager and Kay, 2023), is also interpreted by sociologists as a consequence of socio-economic conditions and parental behaviors (Mahmood and Alwan, 2022). Additionally, parental attitudes and social conditions influence the development of the quest for significance. Specifically, behaviors and rewards valued by parents and others may be internalized by individuals as inherently ‘valuable’ over time. This process of socialization encourages individuals to set high aspirations and strive for exceptional achievements. Cultures that emphasize social mobility are thought to amplify individuals’ ambitions for greatness and recognition (Kruglanski et al., 2022). Turkey, characterized by high social mobility (Özdemir, 2020), may therefore contribute to heightened ambition among individuals. In particular, this social environment provides incentives for emerging adults to engage in a quest for significance. In other words, ambitious emerging adults who seek to achieve a better status and life can accomplish these goals and move toward becoming valuable, important, and recognized individuals.

The stage of emerging adulthood is characterized by intense exploration of identity, career, and romantic relationships, along with significant trial and decision-making efforts (Arnett, 2000). Emerging adults with high levels of ambition may invest considerable effort to achieve their goals, driven by messages of ‘worthiness’ and ‘significance’ from their social environment. When their ambitions align with goals or statuses that are endorsed by themselves, their families, and their communities, they may experience a heightened sense of importance upon achieving them. In other words, the effort they invest in reaching their goals may also contribute to their quest for significance. El Baroudi et al. (2017) reported a positive relationship between ambition and taking responsibility. Consequently, ambitious emerging adults might take on more responsibility than their peers to achieve significance.

In the study, low-level, positive and significant relationships were found between the quest for significance and the impulse (*β* = 0.12) and attitude dimensions (*β* = 0.17) of substance use tendency in emerging adults. These findings support hypotheses H2 and H3. This result is consistent with previous research by Fernandez et al. (2009), Edwards and Neal (2017), Schmidt (2018), Edwards et al. (2021), and Bahl et al. (2023). Individuals from various societal segments who feel marginalized and insignificant may experience increased stress and distress, which can heighten the risk of substance abuse (Flett et al., 2023). As mentioned earlier, emerging adults face numerous stressors (Donat et al., 2019; Metzger et al., 2017; Patterson et al., 2021) and may turn to substance use as a coping mechanism. Elliott (2009) suggests that substance use can serve as an escape from the pain of feeling insignificant to oneself and others. The author also argues that substance use may reflect a lack of self-control and restraint among individuals who do not feel worthy and, therefore, may act as if they have nothing to lose. Consequently, individuals might use substances despite knowing the potential harm.

The loss of significance and the associated threat can trigger the quest for significance (Kruglanski and Bertelsen, 2020; Kruglanski et al., 2014). When an individual experiences feelings of insignificance, they may seek to alleviate these feelings through substance use. Elliott (2009) suggests that insignificance is a painful experience and that individuals might turn to substance use as a means to cope with this pain. Similarly, Lo et al. (2020) noted that interpersonal alienation can lead to substance use, with substances sometimes serving as a ‘soul mate’ during difficult experiences like alienation. Long et al. (2017) found that individuals with social relationship problems tend to have more severe substance use issues. Although a study on emerging adults in Turkey found that participants experienced low levels of alienation (Erol et al., 2018), alienation or problems in social relationships may cause a feeling of insignificance in the individual. Substance use that develops accordingly may act as a kind of preventive for the loss of significance. Consequently, emerging adults may turn to substance use in their quest for significance.

Relationships have been identified between the quest for significance and extremist behaviors (Da Silva et al., 2024; Resta et al., 2023a; Jasko et al., 2020; Kruglanski et al., 2014). Drug use can also be categorized as an extremist behavior. Trucco (2020) suggests that individuals might use substances to socialize with friends and to align their behaviors with those of their chosen peer group. In other words, emerging adults may view substance use as a means to gain significance by fitting in with their social circles and emulating their peers. Thus, substance use behavior may function both as a way to prevent the loss of significance and as an incentive to gain significance. Consequently, a positive and significant relationship may exist between the quest for significance and the impulse and attitude sub-dimensions of substance use behavior in emerging adults.

The study found that the quest for significance mediated the relationship between ambition and the drive dimension of substance use tendency in emerging adults. Similarly, the quest for significance also mediated the relationship between ambition and the attitude dimension of substance use tendency in emerging adults. These results support hypotheses H4 and H5. Entezari and Saroogh Farahan (2013) identified a positive relationship between irrational ambition and substance abuse tendency. Barsukova (2014) describes ambition as a motivational structure representing an individual’s desire for significance and recognition. Ambition drives individuals to seek popularity, fame, and power (Barsukova et al., 2015). In this context, adults with high levels of ambition may turn to substance use as a means to gain significance. Moreover, given that ambitious individuals are more sensitive to the loss of significance through disappointment (Resta et al., 2023a), it can be inferred that those who experience a loss of significance may be more likely to use substances compared to their less ambitious peers. Thus, the quest for significance may serve as a mediating factor between ambition and substance use tendency.

## Limitations and recommendations

This study has several limitations. First, the data were collected using face-to-face and online methods with a convenience sampling approach. Convenience sampling limits the ability to generalize the findings to the broader population (Scholtz, 2021; Turner, 2020). Consequently, the results may not be representative of the emerging adult population in Turkey. Future research should consider using a national sample to increase geographical variability and the generalizability of the findings. Additionally, while this study focused on emerging adults, it is important to note that adolescents are also at risk for substance abuse (Palmer et al., 2009). Future studies could include adolescents to explore the mediating role of the quest for significance in the relationship between ambition and substance abuse tendency in this age group. Another limitation is the cross-sectional design of the study (Kline, 2015). Longitudinal studies could provide more insight into the dynamics of these relationships over time. Lastly, this study employed a quantitative research design. Future research could benefit from qualitative studies to explore these variables in greater depth.

This study identified positive and significant relationships between ambition and the quest for significance, as well as between the quest for significance and substance use tendency in emerging adults. Furthermore, the quest for significance was found to mediate the relationship between ambition and substance use tendency. One key strategy to reduce the risk of substance use in emerging adults is to instill a sense of significance. Mattering, a construct of positive psychology, provides strong protection when individuals feel important to others (Flett et al., 2023). To prevent substance abuse and support recovery, fostering a sense of importance is crucial. Parents can mitigate the risk of substance use by ensuring their children feel valued within the family. Similarly, teachers can protect students by making them feel significant in the school environment. Additionally, school psychological counselors can design and implement preventive programs, particularly for ambitious students, recognizing that they may have an intensified quest for significance and might be at higher risk for substance use as a means to achieve it. In schools, the quest for significance among highly ambitious students can be channeled into positive activities that benefit both the students and their environment, such as joining a basketball team. Teachers and school counselors play a crucial role in guiding students and parents toward these constructive avenues. Adolescents and emerging adults may perceive substance use as a means to gain significance. It is important to communicate to them that substance use is not a legitimate way to achieve significance. Instead, offering alternative opportunities, such as taking dance courses to become a skilled dancer, can help them pursue significance through healthier means. By engaging in these positive activities, adolescents and emerging adults can achieve a sense of significance while avoiding substance use.

## Data Availability

The raw data supporting the conclusions of this article will be made available by the authors, without undue reservation.

## References

[ref1] Alshebami A. S. Alamri M. M. (2020). The role of emotional intelligence in enhancing the ambition level of the students: mediating role of students’ commitment to university. J. Talent Dev. Excell. 12, 2275–2287.

[ref2] Anderson J. C. Gerbing D. W. (1988). Structural equation modeling in practice: a review and recommended two-step approach. Psychol. Bull. 103, 411–423. doi: 10.1037/0033-2909.103.3.411

[ref3] Arnett J. J. (2000). Emerging adulthood: a theory of development from the late teens through the twenties. Am. Psychol. 55, 469–480. doi: 10.1037/0003-066X.55.5.469, PMID: 10842426

[ref4] Arnett J. J. (2005). The developmental context of substance use in emerging adulthood. J. Drug Issues 35, 235–254. doi: 10.1177/002204260503500202

[ref5] Bahl N. Nafstad H. Blakar R. Øversveen E. Brodahl M. Ness O.. (2023). Mattering in older adults in service-assisted recovery processes from substance use problems: conditions, experiences, and implications for action. (Version 1) available at Research Square. doi: 10.21203/rs.3.rs-3041756/v1

[ref6] Barrick M. R. Mount M. K. Li N. (2013). The theory of purposeful work behavior: the role of personality, higher-order goals, and job characteristics. Acad. Manag. Rev. 38, 132–153. doi: 10.5465/amr.2010.0479

[ref7] Barsukova O. V. (2014). Professional ambition: ambition as a motive of professional and career development of person. J. Process Manag. New Technol. Int. 2, 95–98.

[ref8] Barsukova O. V. (2015). Bad ambition. J. Process Manag. New Technol. 3, 8–11.

[ref9] Barsukova O. V. (2016). Psychological characteristics of ambitious person. J. Process Manag. New Technol. 4, 79–80. doi: 10.5937/JPMNT1602079B

[ref10] Barsukova O. V. Mozgovaya N. N. Krishchenko E. P. (2015). The studentsrepresentations of ambition, personal space and trust. Mediterr. J. Soc. Sci. 6, 290–297.

[ref11] Buckner J. D. Ecker A. H. Cohen A. S. (2010). Mental health problems and interest in marijuana treatment among marijuana-using college students. Addict. Behav. 35, 826–833. doi: 10.1016/j.addbeh.2010.04.001 20483200

[ref12] Contu F. Ellenberg M. Kruglanski A. W. Pierro A. (2023). Means substitutability in personal significance restoration. Front. Psychol. 14:1193336. doi: 10.3389/fpsyg.2023.1193336, PMID: 37583600 PMC10423828

[ref13] Creswell J. W. (2014). Research design: Qualitative, quantitative and mixed methods approaches. New York, NY, USA: Sage Publications.

[ref14] Da Silva C. Amadio N. Domingo B. Sarg R. Benbouriche M. (2024). The significance quest theory and the 3N model: a systematic review. Can. Psychol. 65, 58–70. doi: 10.1037/cap0000364

[ref15] de Espíndola M. I. Bedendo A. da Silva E. A. Noto A. R. (2020). Interpersonal relationships and drug use over time among homeless people: a qualitative study. BMC Public Health 20, 1746–1711. doi: 10.1186/s12889-020-09880-2, PMID: 33213421 PMC7678275

[ref16] Donat A. Bilgiç B. Eskiocak A. Koşar D. (2019). Problems and solution suggestions of university students. J. High. Educ. Sci. 9, 451–459. doi: 10.5961/jhes.2019.345, PMID: 39880435

[ref17] Duyan V. Gövebakan R. (2021). “Front Matter,” in Family Matters. İstanbul: Yeni İnsan Publishing House.

[ref18] Edwards K. M. Banyard V. L. Charge L. L. Kollar L. M. M. Fortson B. (2021). Experiences and correlates of violence among American Indian and Alaska native youth: a brief report. J. Interpers. Violence 36, 11808–11821. doi: 10.1177/0886260520983273, PMID: 33371770 PMC8236491

[ref19] Edwards K. M. Neal A. M. (2017). School and community characteristics related to dating violence victimization among high school youth. Psychol. Violence 7, 203–212. doi: 10.1037/vio0000065

[ref20] El Baroudi S. Fleisher C. Khapova S. N. Jansen P. Richardson J. (2017). Ambition at work and career satisfaction: the mediating role of taking charge behavior and the moderating role of pay. Career Dev. Int. 22, 87–102. doi: 10.1108/CDI-07-2016-0124

[ref21] Ellenberg M. Kruglanski A. W. (2024). Power of the network and power from the network: group processes and radicalization. Group Process. Intergroup Relat. 27, 990–1001. doi: 10.1177/13684302241240704, PMID: 39935846

[ref22] Elliott G. C. (ed.). (2009). “Front Matter,” in Family Matters. doi: 10.1002/9781444305784.fmatter

[ref23] Entezari A. Saroogh Farahan R. (2013). The relationship between irrational ambition and tendency to addiction. Sociol. Cult. Stud. 4, 1–21.

[ref24] Erol F. Ziyai N. Y. Karaman D. Tanrıkulu F. Dikmen Y. (2018). Examining the relationship between university students’ alienation levels and social support systems [Oral presentation]. Istanbul, Turkey: ERPA International Congresses on Education. Retrieved from https://d1wqtxts1xzle7.cloudfront.net/63187930/erpa-2018-e-book (Accessed February 07, 2025).

[ref25] Fernandez M. I. Jacobs R. J. Warren J. C. Sanchez J. Bowen G. S. (2009). Drug use and Hispanic men who have sex with men in South Florida: implications for intervention development. AIDS Educ. Prevent. 21, 45–60. doi: 10.1521/aeap.2009.21.5_supp.45, PMID: 19824834

[ref26] Flett G. (2018). The psychology of mattering: Understanding the human need to be significant. London, UK: Academic Press.

[ref27] Flett G. L. Burdo R. Nepon T. (2021). Mattering, insecure attachment, rumination, and self-criticism in distress among university students. Int. J. Ment. Heal. Addict. 19, 1300–1313. doi: 10.1007/s11469-020-00225-z

[ref28] Flett G. L. Casale S. Stoakes A. Nepon T. Su C. (2023). Mattering, substance use, and addictive behaviors: review, analysis, and implications for treatment and prevention. J. Ethn. Subst. Abus. 1-34, 1–34. doi: 10.1080/15332640.2023.2218283, PMID: 37733489

[ref29] Flett G. L. Nepon T. Goldberg J. O. Rose A. L. Atkey S. K. Zaki-Azat J. (2022). The anti-mattering scale: development, psychometric properties and associations with well-being and distress measures in adolescents and emerging adults. J. Psychoeduc. Assess. 40, 37–59. doi: 10.1177/07342829211050544

[ref30] Gray K. M. Squeglia L. M. (2018). Research review: what have we learned about adolescent substance use? J. Child Psychol. Psychiatry 59, 618–627. doi: 10.1111/jcpp.12783, PMID: 28714184 PMC5771977

[ref31] Greenberg J. Roberge M. É. Ho V. T. Rousseau D. M. (2004). “Fairness in idiosyncratic work arrangements: justice as an i-deal” in Research in personnel and human resources management (Leeds, UK: Emerald Group Publishing Limited), 1–34.

[ref32] Hair J. F. Black W. C. Babin B. J. Anderson R. E. (2019). Multivariate data analysis. 8th Edn. London, UK: Cengage Learning.

[ref33] Hirschi A. Spurk D. (2021). Striving for success: towards a refined understanding and measurement of ambition. J. Vocat. Behav. 127:103577. doi: 10.1016/j.jvb.2021.103577

[ref34] Hu L. Bentler P. M. (1999). Cutoff criteria for fit indexes in covariance structure analysis: conventional criteria versus new alternatives. Struct. Equ. Model. Multidiscip. J. 6, 1–55. doi: 10.1080/10705519909540118

[ref35] Jackson K. M. Sher K. J. Schulenberg J. E. (2008). Conjoint developmental trajectories of young adult substance use. Alcohol. Clin. Exp. Res. 32, 723–737. doi: 10.1111/j.1530-0277.2008.00643.x, PMID: 18331376 PMC2705997

[ref36] Jasko K. Webber D. Kruglanski A. W. Gelfand M. Taufiqurrohman M. Hettiarachchi M.. (2020). Social context moderates the effects of quest for significance on violent extremism. J. Pers. Soc. Psychol. 118, 1165–1187. doi: 10.1037/pspi0000198, PMID: 31343222

[ref37] Jones A. B. Sherman R. A. Hogan R. T. (2017). Where is ambition in factor models of personality? Personal. Individ. Differ. 106, 26–31. doi: 10.1016/j.paid.2016.09.057

[ref38] Judge T. A. Kammeyer-Mueller J. D. (2012). On the value of aiming high: the causes and consequences of ambition. J. Appl. Psychol. 97, 758–775. doi: 10.1037/a0028084, PMID: 22545622

[ref39] Keith T. Z. (2019). Multiple regression and beyond: an introduction to multiple regression and structural equation modeling. Routledge. doi: 10.4324/9781315162348

[ref40] Kirst M. Mecredy G. Borland T. Chaiton M. (2014). Predictors of substance use among young adults transitioning away from high school: a narrative review. Subst. Use Misuse 49, 1795–1807. doi: 10.3109/10826084.2014.933240, PMID: 25033376

[ref41] Kline R. B. (2015). The mediation myth. Basic Appl. Soc. Psychol. 37, 202–213. doi: 10.1080/01973533.2015.1049349

[ref42] Kruglanski A. W. Bélanger J. J. Gelfand M. Gunaratna R. Hettiarachchi M. Reinares F.. (2013). Terrorism-a (self) love story: redirecting the significance quest can end violence. Am. Psychol. 68, 559–575. doi: 10.1037/a0032615 24128318

[ref43] Kruglanski A. W. Bertelsen P. (2020). Life psychology and significance quest: a complementary approach to violent extremism and counter-radicalisation. J. Polic., Intell. Count. Terror. 15, 1–22. doi: 10.1080/18335330.2020.1725098

[ref44] Kruglanski A. W. Chen X. Dechesne M. Fishman S. Orehek E. (2009). Fully committed: suicide bombers’ motivation and the quest for personal significance. Polit. Psychol. 30, 331–357. doi: 10.1111/j.1467-9221.2009.00698.x

[ref45] Kruglanski A. W. Ellenberg M. Szumowska E. Molinario E. Speckhard A. Leander N. P.. (2023). Frustration–aggression hypothesis reconsidered: the role of significance quest. Aggress. Behav. 49, 445–468. doi: 10.1002/ab.22092, PMID: 37282763

[ref46] Kruglanski A. W. Gelfand M. J. Bélanger J. J. Hetiarachchi M. Gunaratna R. (2015). “Significance quest theory as the driver of radicalization towards terrorism” in Resilience and resolve: Communities against terrorism, 17–30. doi: 10.1142/9781783267743_0002

[ref47] Kruglanski A. W. Gelfand M. J. Belanger J. J. Sheveland A. Hetiarachchi M. Gunarata R. (2014). The psychology of radicalization and deradicalization: how significance quest impacts violent extremism. Adv. Political Psychol. 35, 69–93. doi: 10.1111/pops.12163

[ref48] Kruglanski A. W. Molinario E. Jasko K. Webber D. Leander N. P. Pierro A. (2022). Significance-quest theory. Perspect. Psychol. Sci. 17, 1050–1071. doi: 10.1177/17456916211034825, PMID: 35133911

[ref49] Lo T. W. Yeung J. W. Tam C. H. (2020). Substance abuse and public health: a multilevel perspective and multiple responses. Int. J. Environ. Res. Public Health 17, 2610–2616. doi: 10.3390/ijerph17072610, PMID: 32290248 PMC7177685

[ref50] Long S. J. Evans R. E. Fletcher A. Hewitt G. Murphy S. Young H.. (2017). Comparison of substance use, subjective well-being and interpersonal relationships among young people in foster care and private households: a cross sectional analysis of the school Health Research network survey in Wales. BMJ Open 7:e014198. doi: 10.1136/bmjopen-2016-014198, PMID: 28219960 PMC5337680

[ref51] Mahmood N. R. Alwan E. H. (2022). The relationship between students' demographic attributes and level of ambition among nursing students. Mosul J. Nur. 10, 279–284. doi: 10.33899/mjn.2022.175578

[ref52] Metzger I. W. Blevins C. Calhoun C. D. Ritchwood T. D. Gilmore A. K. Stewart R.. (2017). An examination of the impact of maladaptive coping on the association between stressor type and alcohol use in college. J. Am. Coll. Heal. 65, 534–541. doi: 10.1080/07448481.2017.1351445, PMID: 28708021 PMC6134834

[ref53] Mochrie K. D. Whited M. C. Cellucci T. Freeman T. Corson A. T. (2020). ADHD, depression, and substance abuse risk among beginning college students. J. Am. Coll. Heal. 68, 6–10. doi: 10.1080/07448481.2018.1515754, PMID: 30257141

[ref54] NCDAS (2020). Drug use among youth: Facts & statistics. Available online at: https://drugabusestatistics.org/teen-drug-use/ (July 18, 2024).

[ref55] Önder M. S. (2023). Substance initiation tendency scale (SITS): validity and reliability study. Subst. Use Misuse 58, 578–584. doi: 10.1080/10826084.2023.2177971, PMID: 36762469

[ref56] Özdemir C. (2020). Measurement of social stratification and mobility in Turkey through occupations. J. Econ. Cul. Soc. 1, 59–77. doi: 10.26650/JECS2019-0053, PMID: 38418444

[ref57] Palmer R. H. C. Young S. E. Hopfer C. J. Corley R. P. Stallings M. C. Crowley T. J.. (2009). Developmental epidemiology of drug use and abuse in adolescence and young adulthood: evidence of generalized risk. Drug Alcohol Depend. 102, 78–87. doi: 10.1016/j.drugalcdep.2009.01.012, PMID: 19250776 PMC2746112

[ref58] Patterson Z. R. Gabrys R. L. Prowse R. K. Abizaid A. B. Hellemans K. G. McQuaid R. J. (2021). The influence of COVID-19 on stress, substance use, and mental health among postsecondary students. Emerg. Adulthood 9, 516–530. doi: 10.1177/21676968211014080

[ref59] Pettigrove G. (2007). Ambitions. Ethical Theory Moral Pract 10, 53–68. doi: 10.1007/s10677-006-9044-4

[ref60] Resta E. Ellenberg M. Kruglanski A. W. Pierro A. (2022). Marie curie vs. Serena Williams: ambition leads to extremism through obsessive (but not harmonious) passion. Motiv. Emot. 46, 382–393. doi: 10.1007/s11031-022-09936-3

[ref61] Resta E. Ellenberg M. Kruglanski A. W. Pierro A. (2023a). Ambition and extreme behavior: relative deprivation leads ambitious individuals to self-sacrifice. Front. Psychol. 14:1108006. doi: 10.3389/fpsyg.2023.1108006, PMID: 37502752 PMC10370493

[ref62] Resta E. Kruglanski A. W. Ellenberg M. Pierro A. (2023b). Ambition-driven aggression in response to significance-threatening frustration. Eur. J. Soc. Psychol. 53, 1458–1474. doi: 10.1002/ejsp.2988

[ref63] Rosenberg M. McCullough B. C. (1981). Mattering: inferred significance and mental health among adolescents. Res. Community Ment. Health 2, 163–182.

[ref64] Şahin E. S. Ayvaz A. (2024). Adaptation of ambition scale (AS) to Turkish culture [Oral presentation]. 5th International 5th. Turkey: January Social Sciences and Humanities Congress Adana.

[ref65] Şahin E. S. Derin S. (2023). Development, validity and reliability of significance quest scale (SQS). Int. J. Educ. Res. Rev. 8, 446–458. doi: 10.24331/ijere.1226598

[ref66] SAMHSA (2021). Results from the 2021 National Survey on drug use and health: Summary of national findings. Available online at: https://datatools.samhsa.gov/saes/state (Accessed July 18, 2024).

[ref67] Schmidt C. (2018). Examining the role of interpersonal and societal mattering in the health and wellbeing of rural adolescents [Unpublished doctoral dissertation]. Michigan, USA: University of Michigan.

[ref68] Scholtz S. E. (2021). Sacrifice is a step beyond convenience: a review of convenience sampling in psychological research in Africa. SA J. Ind. Psychol. 47, 1–12. doi: 10.4102/sajip.v47i0.1837, PMID: 39507825

[ref69] Schumpe B. M. Bélanger J. J. Moyano M. Nisa C. F. (2020). The role of sensation seeking in political violence: an extension of the significance quest theory. J. Pers. Soc. Psychol. 118, 743–761. doi: 10.1037/pspp0000223 30382738

[ref70] Security General Directorate (2018). Survey on attitudes and behaviors towards tobacco, alcohol and substance use in the general population in Turkey. Available online at: https://www.narkotik.pol.tr/kurumlar/narkotik.pol.tr/Duyurular/ (Accessed July 18, 2024).

[ref71] Skidmore C. R. Kaufman E. A. Crowell S. E. (2016). Substance use among college students. Child Adolescent Psychiatric Clinics 25, 735–753. doi: 10.1016/j.chc.2016.06.004, PMID: 27613349

[ref72] Taylor J. Turner R. J. (2001). A longitudinal study of the role and significance of mattering to others for depressive symptoms. J. Health Soc. Behav. 42:310. doi: 10.2307/3090217, PMID: 11668776

[ref73] Tibbe T. D. Montoya A. K. (2022). Correcting the bias correction for the bootstrap confidence interval in mediation analysis. Front. Psychol. 13, 1–21. doi: 10.3389/fpsyg.2022.810258, PMID: 35712166 PMC9197131

[ref0090] Trucco E. M. (2020). A review of psychosocial factors linked to adolescent substance use. Pharmacol. Biochem. Behav. 196:172969. doi: 10.1016/j.pbb.2020.172969 32565241 PMC7415605

[ref74] TURKSTAT (2022). Turkey health survey. Available online at: https://data.tuik.gov.tr/Bulten/Index?p=Turkiye-Saglik-Arastirmasi-2022-49747 (Accessed July 18, 2024).

[ref75] TURKSTAT (2025). Youth in statistics, 2023. Available online at: https://data.tuik.gov.tr/Bulten/Index?p=Istatistiklerle-Genclik-2023-53677 on 06.02.2025 (Accessed February 06, 2024).

[ref76] Turner D. P. (2020). Sampling methods in research design. Headache 60, 8–12. doi: 10.1111/head.13707 31913516

[ref77] Walters K. S. Bulmer S. M. Troiano P. F. Obiaka U. Bonhomme R. (2018). Substance use, anxiety, and depressive symptoms among college students. J. Child Adolesc. Subst. Abuse 27, 103–111. doi: 10.1080/1067828X.2017.1420507

[ref78] Webber D. Klein K. Kruglanski A. Brizi A. Merari A. (2017). Divergent paths to martyrdom and significance among suicide attackers. Terror. Political Viol. 29, 852–874. doi: 10.1080/09546553.2015.1075979

[ref79] Welsh J. W. Shentu Y. Sarvey D. B. (2019). Substance use among college students. FOCUS J. Am. Psychiatric Assoc. 17, 117–127. doi: 10.1176/appi.focus.20180037, PMID: 31975967 PMC6527004

[ref80] Yager J. Kay J. (2023). Ambition and ıts psychopathologies. J. Nerv. Ment. Dis. 211, 257–265. doi: 10.1097/NMD.0000000000001644, PMID: 36975544

[ref81] Yarnell S. Li L. MacGrory B. Trevisan L. Kirwin P. (2020). Substance use disorders in later life: a review and synthesis of the literature of an emerging public health concern. Am. J. Geriatr. Psychiatry 28, 226–236. doi: 10.1016/j.jagp.2019.06.005, PMID: 31340887

[ref82] Zhang H. Kyzar E. J. Bohnsack J. P. Kokare D. M. Teppen T. Pandey S. C. (2018). Adolescent alcohol exposure epigenetically regulates CREB signaling in the adult amygdala. Sci. Rep. 8:10376. doi: 10.1038/s41598-018-28415-9, PMID: 29991681 PMC6039491

